# Value of Axillary Palpation and Ultrasound, and Axillary Recurrence in Male Breast Cancer: Results of the Registry Study

**DOI:** 10.1002/ijc.70658

**Published:** 2026-07-16

**Authors:** Monika Graeser, Susen Bothin, Oleg Gluz, Mathias Warm, Atanas Ignatov, Holm Eggemann

**Affiliations:** ^1^ West German Study Group Moenchengladbach Germany; ^2^ Breast Center Niederrhein, Ev. Hospital Bethesda Moenchengladbach Germany; ^3^ Department of Obstetrics and Gynecology University Witten/Herdecke Witten Germany; ^4^ Department of Gynecology and Obstetrics Klinikum Magdeburg Magdeburg Germany; ^5^ University Clinics Cologne Cologne Germany; ^6^ Breast Center, Municipal Hospital Holweide Cologne Germany; ^7^ Department of Gynaecology and Obstetrics Otto‐von‐Guericke University Magdeburg Germany

**Keywords:** axilla, breast neoplasms, lymphatic metastasis, male, palpation, ultrasonography

## Abstract

Clinical and sonographic examination of breasts and axillae is standard in breast cancer (BC) care. The aim of this study is to highlight the value of axillary ultrasound in identifying axillary recurrences in male BC, which is still little considered in the literature. This was a registry‐based observational multicenter cohort study in Germany. Five hundred and seventy‐seven male patients with invasive non‐metastatic BC were included. Five hundred and sixty three patients underwent a clinical examination of the axilla, and 500 had an axillary ultrasound. The examination results were used to determine sensitivity and specificity for predicting lymph node metastases depending on the nodal status. The axillary recurrences were determined during follow‐up. The sensitivity and specificity were 30.4% and 93.2% for axillary palpation, 42.2% and 88.9% for ultrasound, and 49.5% and 85.3% for both methods combined. Both methods, considered either separately or combined, showed an increase in sensitivity with increasing lymph node involvement. In the combined analysis, the sensitivity varied from 38.5% (pN1) to 80.0% (pN3). Sensitivity was higher in Grade 3 compared to Grades 1 or 2 carcinomas, in patients aged > 68 years compared to younger patients, and in non‐obese compared to obese patients (BMI > 30 kg/m^2^). The axillary recurrence rate was 1.21%. The axillary examination is an important part of pre‐therapeutic diagnostics in male patients; however, ultrasound and palpation appear less sensitive than in female disease. The probability of axillary recurrence is very low after adequate therapy in male BC patients without distant metastases and in those with a low risk of metastases based on tumor biology.

## What's New?

The rarity of male breast cancer has limited research on disease‐specific management, particularly the assessment of axillary lymph nodes, an important prognostic factor. In this study, data on male invasive nonmetastatic breast cancer was evaluated to determine the accuracy of axillary palpation and ultrasound for detecting lymph node metastases and to assess axillary recurrence. Analyses show that ultrasound is more sensitive than palpation, and combining both methods further improves detection. Sensitivity, however, remained lower than reported in women. Post‐treatment axillary recurrence was rare. The findings support tailored diagnostic strategies to improve staging and management in men with early breast cancer.

## Graphical abstract

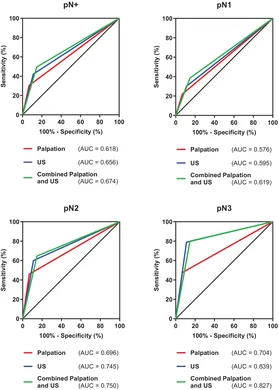

**Abbreviations**
ALND: axillary lymph node dissection
AUC: area under the curve
BMI: body mass index
DCIS: ductal carcinoma in situ
ER: estrogen receptor
HER2: human epidermal growth factor receptor 2
NPV: negative predictive value
NST: no special type
PPV: positive predictive value
PR: progesterone receptor
ROC: receiver operating characteristic
SENS: sensitivity
SNB: sentinel node biopsy
SPEC: specificity
US: ultrasonography

## 1 Introduction

Male breast cancer is a rare disease, accounting for less than 1% of all male tumors [1]. Similar to female breast cancer, the incidence is increasing [2]. According to the German Cancer Registry, there were 740 new cases in 2020, which resulted in a standardized incidence rate of 1.1 per 100,000 people [3]. Due to the rarity of male breast cancer, there is a substantial knowledge gap in the management of the disease. The most recent guidelines for male breast cancer is based on the diagnosis, treatment, and follow‐up of the disease in postmenopausal women, despite differences in clinical and molecular characteristics [4, 5, 6]. Compared to women, male breast cancer is generally diagnosed at a later stage and demonstrates more advanced features. Additionally, a higher percentage of male than female breast cancers are classified as infiltrating ductal carcinoma, express estrogen (ER) and progesterone receptors (PR), and are predominantly luminal A‐like and luminal B‐like [7, 8, 9]. Due to these clinical and molecular differences, male breast cancer patients are at a higher risk of recurrence and have worse long‐term outcomes than females [10, 11, 12, 13]. Additionally, one study attributed lower survival rates to undertreatment in male patients [10].

Finally, there is limited data on treatment efficacy due to the fact that males are underrepresented in breast cancer trials. It has been shown that males comprise 0.42% of patients in clinical trials in breast cancer, which is less compared to the incidence of male breast cancer, accounting for approximately 1% of all breast cancer diagnoses [14].

Given the above, male breast cancer presents unique challenges, particularly concerning the assessment of axillary lymph nodes, which are critical for staging and treatment planning. The involvement of the axilla is a significant prognostic factor, influencing both local and systemic therapy decisions. For instance, male breast cancer tends to present at a more advanced stage compared to female breast cancer, with a higher likelihood of lymph node involvement at diagnosis [15]. The usual methods of axillary diagnostics—clinical examination and sonography—for female breast cancer have not yet been sufficiently investigated for male breast cancer. In this study, we evaluated the accuracy of palpation and ultrasound for the detection of axillary lymph node metastases and assessed the axillary recurrences in male breast cancer patients.

## 2 Patients and Methods

This was a German national prospective cancer registry study (DRKS00009536) compiling data from male breast cancer patients collected in 136 German gynecological clinics between December 2007 and December 2022.

The primary objective of the study was to prospectively recognize the primary cases of breast cancer in men and determine recurrence‐free survival. The secondary objectives included determining overall survival, stage and biological characteristics, validation of therapeutic modalities, sensitivity and specificity of diagnosis, and analysis of histopathological characteristics in tumor tissue.

Male patients aged ≥ 18 years with histologically confirmed primary breast cancer were included. Exclusion criteria for the evaluation were the performance of neoadjuvant chemotherapy, the presence of ductal carcinoma in situ, M1 status, or MX with an increased risk of metastasis (N+, T > 2, human epidermal growth factor receptor 2 (HER2)‐positive status, triple‐negative breast cancer), and the lack of documentation of both axillary palpation and sonography. Adjuvant systemic therapies and radiation were performed when indicated. The patients were observed every 6 months in a 5‐year follow‐up. Axillary recurrences were identified, and the cases were described.

### 2.1 Examinations

An experienced gynecologist carried out the clinical and sonographic examination of the breast and axilla. Questions about palpable axillary and sonographically suspicious lymph nodes were answered with “yes” (= examination positive) or “no” (= examination negative). The material obtained intraoperatively was examined by histopathology, and the pathological nodal status was determined.

### 2.2 Statistical Analysis

The statistical calculations were performed using the IBM SPSS 28.0 software. Frequency analyses of patient and tumor characteristics, as well as therapies, were performed. The value of palpation and ultrasound in estimating the postoperative pathological nodal status was analyzed by calculating sensitivity, specificity, and positive and negative predictive values. The chi‐square test was used to determine the sensitivity and specificity of the axillary palpation and axillary ultrasound. The significance level was a *p* value of 0.05. Receiver operating characteristic (ROC) curves and the ROC area under the curve (AUC) for predicting a pathological nodal status by palpation and ultrasound were computed. Additionally, these characteristics were analyzed in combination with palpation and ultrasonography, where at least one examination yielded a positive result.

## 3 Results

Out of 997 identified male breast cancer patients, 420 patients were excluded due to M1 status or MX with an increased risk of metastasis (*n* = 334), DCIS (*n* = 24), neoadjuvant chemotherapy (*n* = 48), and neither axillary palpation nor sonography documented (*n* = 14). The remaining 577 patients met the inclusion criteria and were included in the study. The clinical, histopathological, and therapeutic characteristics of the patient collective are shown in Table 1. The mean age at diagnosis was 68 years (range 26 to 96), and the mean body mass index (BMI) was 28.5 kg/m^2^ (range 16.6–62.5). The histological carcinoma type was no special type in 518 cases (90.5%), of which 33 cases (5.7%) had an additional histological component. Ten cases (1.7%) showed an invasive papillary carcinoma, 8 cases (1.4%) an invasive lobular carcinoma, and 35 cases (6.1%) another histological type. Five hundred and sixty‐five tumors (98.1%) were ER‐positive, 533 (93.0%) PR‐positive, and 44 tumors (9.0%) were HER2‐positive. Two hundred and thirty tumors (40.1%) were classified as pT1, 268 (46.7%) as pT2, 12 (2.1%) as pT3 and 64 (11.1%) as pT4. Three hundred and thirty‐three participants (59.5%) were classified as pN0, 156 (27.9%) as pN1, 50 (8.9%) as pN2 and 21 (3.8%) as pN3. The grading showed the following distribution: G1 in 51 patients (8.9%), G2 in 392 patients (68.5%), and G3 in 129 patients (22.6%). Most often, patients underwent sentinel node biopsy alone (*n* = 305, 59.3%), axillary dissection alone (*n* = 158, 30.7%), and sentinel node biopsy followed by axillary dissection (*n* = 51, 9.9%). Five hundred and forty‐six patients (96.6%) and only 19 patients had breast‐conserving surgery (3.4%).

**TABLE 1:** Patients' characteristics.

Characteristics		All patients (*N* = 577)
Age at diagnosis in years, mean (min‐max)		68.27 (26–96)
Height in cm, mean (min‐max)		176.9 (154–203)
Weight in kg, mean (min‐max)		89.3 (51–185)
BMI in kg/m^2^, mean (min‐max)		28.5 (16.6–62.5)
Histological type, *n* (%)	NST	485 (86.0)
	NST + DCIS	26 (4.5)
	Invasive papillary	10 (1.7)
	Invasive lobular	8 (1.4)
	Invasive micropapillary	6 (1.0)
	Cribriform	5 (0.9)
	NST + invasive lobular	4 (0.7)
	Mucinous	4 (0.7)
	Invasive solid papillary	3 (0.5)
	Mucinous + NST	2 (0.3)
	Invasive with medullary pattern	1 (0.2)
	Micropapillary + NST	1 (0.2)
	Other	9 (1.6)
	No information	13
pT, *n* (%)	1	230 (40.1)
	2	268 (46.7)
	3	12 (2.1)
	4	64 (11.1)
	No information	3
pN, *n* (%)	0	333 (59.5)
	1	156 (27.9)
	2	50 (8.9)
	3	21 (3.8)
	No information	17
M, *n* (%)	0	549 (96.8)
	X	18 (3.2)
	No information	10
Grade, *n* (%)	1	51 (8.9)
	2	392 (68.5)
	3	129 (22.6)
	No information	5
L, *n* (%)	0	336 (61.1)
	1	214 (38.9)
	No information	27
V, *n* (%)	0	520 (95.4)
	1	25 (4.6)
	No information	32
ER status, *n* (%)	Positive	565 (98.1)
	Negative	11 (1.9)
	No information	1
PR status, *n* (%)	Positive	533 (93.0)
	Negative	40 (7.0)
	No information	4
HER2/neu status, *n* (%)	Positive	44 (9.0)
	Negative	443 (91.0)
	No information	90
Axillary palpation result, *n* (%)	Positive	94 (16.7)
	Negative	469 (83.3)
	No information	14
Axillary ultrasonography result, *n* (%)	Positive	118 (23.6)
	Negative	382 (76.4)
	No information	77
Breast cancer surgery, *n* (%)	Mastectomy	546 (96.6)
	Breast‐conserving surgery	19 (3.4)
	No information	12
Axillary surgery, *n* (%)	ALND	158 (30.7)
	SNB	305 (59.3)
	SNB + ALND	51 (9.9)
	No information	63
Chemotherapy, *n* (%)	Yes	197 (35.6)
	No	356 (64.4)
	No information	24
Radiotherapy, *n* (%)	Yes	268 (47.4)
	No	297 (52.6)
	No information	12

Abbreviations: ALND, axillary lymph node dissection; DCIS, ductal carcinoma in situ; NST, no special type; SNB, sentinel lymph node biopsy.

Palpation of the axilla was performed in 563 patients, and an axillary ultrasound was performed in 500 patients; 486 patients had both axillary palpation and axillary ultrasound. In relation to the postoperative pathological nodal status, palpation showed a 30.4% sensitivity and a 93.2% specificity (chi‐square test *p* < 0.05, Table 2). The sensitivity of axillary ultrasonography was 42.2%, with a specificity of 88.9%. A combined analysis of palpation and ultrasonography, where at least one examination yielded a positive result, demonstrated a sensitivity of 49.5% and a specificity of 85.3%. The false negative rate (FNR) was 69.6% for palpation, 57.8% for ultrasonography, and 50.5% for combined examination.

**TABLE 2:** Sensitivity and specificity of axillary assessments.

Axillary assessment	SPEC in pN+	SENS in pN+	SENS in pN1	SENS in pN2	SENS in pN3	FNR in pN+	FNR in pN1	FNR in pN2	FNR in pN3	Prevalence	PPV	NPV
Palpation	93.2%	30.4%	22.0%	46.0%	47.6%	69.6%	78.0%	54.0%	52.4%	40.95%	77.8%	65.9%
US	88.9%	42.2%	30.1%	60.0%	78.9%	57.8%	69.9%	40.0%	21.1%	40.95%	72.5%	68.9%
Palpation and US combined	85.3%	49.5%	38.5%	64.6%	80.0%	50.5%	61.5%	35.4%	20.0%	41.96%	70.9%	70.0%

Abbreviations: FNR, false‐negative rate; NPV, negative predictive value; PPV, positive predictive value; SENS, sensitivity; SPEC, specificity; US, ultrasonography.

The sensitivity of examinations increased with the lymph node involvement from pN1 to pN3: from 22.0% to 47.6% for axillary palpation, and from 30.1% to 78.9% for ultrasound, and from 38.5% to 80.0% for the combination of both methods (Table 2). As expected, this was accompanied by an FNR decrease with lymph node involvement, particularly for ultrasonography. Overall, the accuracy was highest for the combined assessment (AUC = 0.674), followed by ultrasound (AUC = 0.656) and palpation (AUC = 0.618, Figure 1). The accuracy of examinations (either individually or combined) improved with increasing nodal status.

**FIGURE 1:**
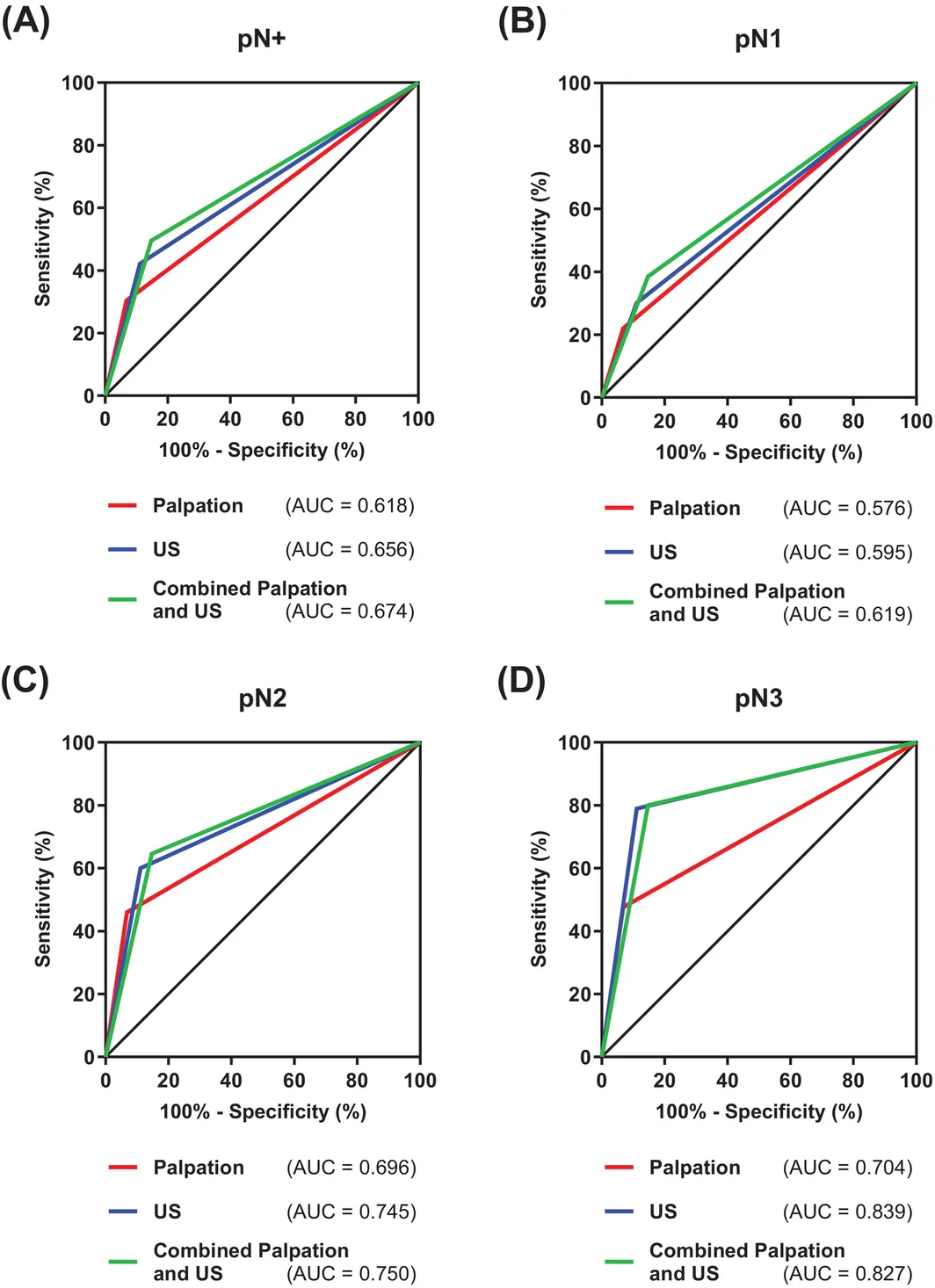
Receiver operating characteristic curves and area under the curve values for axillary palpation, ultrasound, or a combination of both methods. Data are shown for all patients with node‐positive disease (A), and for pN1 (B), pN2 (C), and pN3 cases (D).

Table 3 shows the sensitivities and specificities of palpation and ultrasound depending on the age at the onset of the disease, BMI, and tumor grade. The specificity of assessments was comparable in analysis by age at diagnosis (≤ 68 vs. > 68 years) and tumor grade (G1, G2 vs. G3); however, it was higher in patients with BMI ≤ 30 versus > 30 kg/m^2^. The overall sensitivity of assessments (individual or combination) was higher in patients diagnosed at > 68 years and in those with G3 tumors. In contrast, there was no apparent difference in sensitivity according to BMI when either method was used separately. The sensitivity of assessments in all subgroups increased with a higher lymph node involvement; however, note that there were fewer than 5 patients with pN3 disease in some subgroups (age ≤ 68 years, BMI > 30 kg/m^2^, G3 tumors), and thus, these results should be interpreted with caution. Overall, ultrasound yielded higher sensitivity than palpation, particularly in the subgroup aged ≤ 68 years (independently of the nodal status) and in the G1‐G2 subgroup with pN1 disease. Furthermore, the gains in sensitivity from adding palpation to ultrasound were minimal in the pN2 disease compared to ultrasound alone. However, a combined analysis in pN1 disease increased sensitivity compared to either method alone, particularly in the subgroup with G3 tumors, those with a BMI ≤ 30 kg/m^2^, and in patients aged > 68 years.

**TABLE 3:** Sensitivity and specificity of axillary assessments by age at diagnosis, BMI, and tumor grade.

Axillary assessment	SPEC	SENS in pN+	SENS in pN1	SENS in pN2	SENS in pN3
Analysis by age at diagnosis					
Palpation					
≤ 68 years	94.0%	23.4%	16.9%	30.8%	50.0%^a^
> 68	93.0%	37.8%	27.4%	62.5%	46.2%
US					
≤ 68 years	88.6%	37.3%	26.1%	54.2%	75.0%
> 68	89.0%	48.4%	35.0%	66.7%	81.8%
Palpation and US combined					
≤ 68 years	84.7%	43.0%	32.3%	56.0%	75.0%
> 68	86.2%	56.4%	44.6%	73.9%	83.3%
Analysis by BMI					
Palpation					
BMI ≤ 30 kg/m^2^	97.2%	30.8%	22.9%	47.1%	40.0%
BMI > 30 kg/m^2^	84.2%	29.0%	19.1%	46.7%	60.0%^a^
US					
BMI ≤ 30 kg/m^2^	90.6%	43.7%	30.5%	63.3%	78.6%
BMI > 30 kg/m^2^	84.3%	40.6%	27.9%	57.1%	80.0%^a^
Palpation and US combined					
BMI ≤ 30 kg/m^2^	88.8%	52.6%	41.2%	69.7%	78.6%
BMI > 30 kg/m^2^	76.5%	42.9%	31.0%	57.1%	80.0%^a^
Analysis by tumor grade					
Palpation					
G1, G2	92.5%	27.5%	19.3%	42.9%	43.8%
G3	95.5%	39.3%	31.4%	53.5%	60.0%^a^
US					
G1, G2	89.0%	40.4%	30.7%	53.5%	78.6%
G3	87.9%	48.1%	29.0%	73.3%	80.0%
Palpation and US combined					
G1, G2	85.0%	47.0%	36.6%	60.6%	80.0%
G3	85.7%	57.4%	45.5%	73.3%	80.0%^a^

Abbreviations: SENS, sensitivity; SPEC, specificity; US, ultrasonography.

^a^ < 5 cases.

Out of eight purely invasive lobular carcinomas, two (25%) showed a positive lymph node status (pN1 and pN3, respectively). Compared to the overall population, in which 40.8% had lymph node involvement, this represents a 15.8% lower rate of nodal positivity among the purely lobular carcinomas. In the case with pN1, the positive lymph node status was not detected either by palpation or ultrasound. In the case with pN3, palpation was suspicious, but no ultrasound documentation was available.

Among the 529 patients with available follow‐up, 7 participants (1.2%) had axillary recurrence by December 2022: three recurrences occurred after 12 months of follow‐up, and one each after 18, 36, 42, and 60 months (Table 4). At the last documented follow‐up, six patients were alive, and one participant had died. The mean age of onset in patients with axillary recurrence was 63.4 years (range 37–75 years). These seven patients had a mean BMI of 27.3 kg/m^2^ (range 21.4–37.4 kg/m^2^). All cases were of no special type histology and were ER‐ and PR‐positive; one case was HER2‐positive. Three cases were G3 tumors, four were pT2, and one was classified as pN1 (not detected in the axillary examination). All seven patients underwent a mastectomy; five of them had a sentinel node biopsy, and one had an axillary dissection.

**TABLE 4:** Characteristics of cases with axillary recurrence.

Characteristics		Axillary recurrence (*n* = 7)
Time from diagnosis till axillary recurrence in months, *n* (%)	12	3 (42.9)
	18	1 (14.3)
	36	1 (14.3)
	42	1 (14.3)
	60	1 (14.3)
Status at last follow‐up, *n* (%)	Alive	6 (85.7)
	Dead	1 (14.3)
Age at diagnosis in years, mean (min‐max)		63.4 (37–75)
Height in cm, mean (min‐max)		180.1 (173–188)
Weight in kg, mean (min‐max)		88.1 (68–112)
BMI in kg/m^2^, mean (min‐max)		27.3 (21.4–37.4)
Histological type, *n* (%)	NST	7 (100.0)
pT, *n* (%)	1 2	3 (42.9) 4 (57.1)
pN, *n* (%)	0	5 (83.3)
	1	1 (16.7)
	Missing	1
M, *n* (%)	0	5 (100.0)
	Missing	2
Grade, *n* (%)	2	4 (57.1)
	3	3 (42.9)
L, *n* (%)	0	4 (57.1)
	1	3 (42.9)
V, *n* (%)	0	7 (100.0)
ER status, *n* (%)	Positive	7 (100.0)
PR status, *n* (%)	Positive	7 (100.0)
HER2/neu status, *n* (%)	Positive	1 (14.3)
	Negative	6 (85.7)
Axillary palpation result, *n* (%)	Positive	1 (14.3)
	Negative	6 (85.7)
Axillary ultrasonography result, *n* (%)	Positive	1 (14.3)
	Negative	6 (85.7)
Breast cancer surgery, *n* (%)	Mastectomy	7 (100.0)
Axillary surgery, *n* (%)	Axillary dissection	1 (16.7)
	SNB	5 (83.3)
	Missing	1
Chemotherapy, *n* (%)	Yes	2 (28.6)
	No	5 (71.4)
Radiotherapy, *n* (%)	Yes	2 (28.6)
	No	5 (71.4)

Abbreviations: NST, no special type; sentinel lymph node biopsy.

## 4 Discussion

While there are several studies on axillary diagnostics in female breast cancer, data from larger cohorts for male breast cancer are lacking. In our study of 577 male patients, we found that ultrasound yielded better sensitivity than palpation for detecting axillary lymph node metastases, while both methods had a similar specificity. The sensitivity of ultrasound increased with lymph node involvement, and it was only marginally improved by combining it with palpation in pN2 and pN3 disease. In contrast, we observed that a combination of both methods resulted in a noticeable increase in sensitivity for pN1 disease. Furthermore, the sensitivity of palpation was particularly poor in patients aged 68 years or younger and in those with G1 or G2 tumors. Overall, these results indicate that ultrasound is a preferable method for assessment of the axilla, and the diagnostic workup in male breast cancer should not rely only on palpation.

Compared with our analysis, studies in female breast cancer have shown a higher sensitivity of axillary ultrasound, ranging from approximately 50%–70% [16, 17, 18, 19, 20, 21]. For instance, a meta‐analysis in 10,374 patients demonstrated a sensitivity and specificity of 55% and 99% for axillary ultrasound [22]. This indicates that affected lymph nodes are apparently less likely to be detected in male than in female breast cancer patients. Furthermore, compared with published female breast cancer cohorts, our male cohort showed similar FNR for palpation (approximately 70% in both sexes), but higher FNR for axillary ultrasound (approximately 60% vs. 45%) and for the combination of both methods (approximately 50% vs. 40%) [21, 23]. Therefore, there is potentially a higher risk of missing nodal metastases with these assessment methods in men than in women, particularly with regard to the ultrasound. There are several potential reasons for the variability of sensitivities, specificities, and FNR between the studies performed in male and female breast cancer. Aside from interobserver variability and differences in assessment protocols, which could affect the comparison of ultrasound accuracy between studies, differences in clinical and biological characteristics between the sexes may also play a role. For example, a vast majority of male breast cancers are of luminal A or luminal B type, and this type of breast cancer was shown to negatively affect the sensitivity of axillary ultrasound in female disease [24]. With an overall sensitivity of 42.2%, ultrasound appears to be better than palpation (30.4%) for assessing the axilla in male patients, and its sensitivity could be improved to 49.5% by combining both methods. Our data thus indicate that axillary sonography with a sensitivity of 42.2% is an effective tool for axillary staging and should also be a standard imaging approach in male breast cancer in order to plan further treatment. However, ultrasound diagnostics are inadequate to exclude axillary involvement and should be followed by a routine histological examination of the axilla. Collectively, our data show that while ultrasound, both on its own and in combination with palpation, has potential, there is ample opportunity for enhancement. This highlights the need for the development of more optimal diagnostic methods.

Our study is one of the first large analyses of the axillary recurrence in male breast cancer. We observed that the probability of axillary recurrence after guideline‐compliant treatment is very low at 1.21%, and it is comparable with female disease (0.1%– 8%) [25, 26, 27]. Nevertheless, it is necessary to acknowledge the differences in inclusion criteria between the studies, which could affect the recurrence rates, for example, the inclusion of patients with negative sentinel lymph nodes or only those undergoing axillary lymph node dissection. Studies comparing survival according to sex found that male patients have worse disease‐free survival, while overall survival was similar [24, 28]. However, other studies have demonstrated a worse overall survival in male patients with early‐stage breast cancer [10, 29]. For instance, a 2024 population‐based cohort study revealed 5‐year overall survival rates of 81.5% in males versus 92.8% in females with stage I disease [13]. Several studies explored the factors associated with survival in male breast cancer. For example, Eggemann et al. demonstrated that patients who had tamoxifen therapy had significantly longer disease‐free survival than those without such treatment [30]. Furthermore, Chung et al. found that axillary lymph node dissection is associated with improved overall survival in male patients compared to sentinel lymph node biopsy [31]. Finally, several clinical factors have been identified to be potentially associated with poorer survival in male compared to female breast cancer, including a higher proportion of advanced stages of disease and lower rates of adjuvant therapy in male patients [32]. Interestingly, male breast cancer patients exhibit distinct genetic alterations, including a higher frequency of mutations in ERBB2 and PIK3CA genes, that could potentially impair the efficacy of targeted treatments and lead to poorer outcomes than in female patients [33].

Our study has limitations. For example, the study excluded patients with MX status and an increased risk of metastasis (e.g., N+, T > 2, HER2‐positive or triple‐negative disease). These patients, together with M1 cases, accounted for approximately one‐third of all screened patients. Computer tomography (CT) is a standard diagnostic workup in Germany; however, we did not collect CT data and thus cannot report the number of patients without CT or with MX status plus increased risks of metastasis. Because N+, T > 2, HER2‐positive or triple‐negative disease features are associated with a higher likelihood of nodal involvement, the analyzed cohort is likely enriched for patients with more favorable disease biology and/or earlier stage. Consequently, this selection may have led to an underestimation of nodal positivity and axillary recurrence. In the sensitivity and specificity analysis of nodal status, several subgroups of patients with pN3 disease (age ≤ 68 years, BMI > 30 kg/m^2^, G3 tumors) had fewer than five cases. This limits the statistical reliability of sensitivity estimates in these small subgroups, and these results should not be overinterpreted. Due to the very low number of events, axillary recurrence was reported as event counts. Furthermore, follow‐up duration was unavailable due to erroneous date entries in the site documentation. Together, these limitations precluded meaningful time‐to‐event and recurrence risk factor analyses; therefore, the recurrence estimate should be interpreted with caution. Finally, our findings may not fully generalize to non‐German healthcare settings. Therefore, our estimates of the diagnostic performance of axillary palpation and ultrasound, as well as the observed very low axillary recurrence rate, may not be fully generalizable to higher‐risk male breast cancer populations, and the recurrence risk in an unselected cohort could be higher.

## 5 Conclusions

Our results indicate that axillary ultrasound is more sensitive than palpation for the prediction of lymph node metastasis in male breast cancer. However, both methods (separately or combined) appear less sensitive than in female disease, which stresses the need for better methods for examining the axilla in male patients. The probability of axillary recurrence is very low after adequate therapy in male breast cancer patients without distant metastases and in those with a low risk of metastases based on tumor biology.

## Author Contributions

**Monika Graeser:** conceptualization, investigation, writing – original draft, writing – review and editing, formal analysis. **Susen Bothin:** conceptualization, writing – original draft, writing – review and editing, formal analysis. **Oleg Gluz:** conceptualization, investigation, writing – review and editing. **Mathias Warm:** writing – review and editing, investigation. **Atanas Ignatov:** writing – review and editing, investigation. **Holm Eggemann:** writing – review and editing, investigation.

## Ethics Statement

The study was approved by the Ethics Committee at Otto von Guericke University, Magdeburg, Germany (approval number: 114/09). All patients provided written informed consent.

## Conflicts of Interest

O.G. reports potential financial conflicts of interest related to honoraria from Gilead, Roche, Exact Sciences, MSD, AstraZeneca, Daiichi Sankyo, Novartis, Pfizer, and Lilly. All other authors declare no conflicts of interest.

## Data Availability

Deidentified participant data will be made available upon a reasonable request to the corresponding author.
